# Supplementary material for: Are carbon emissions trading and green financial instruments synergistic? -Comprehensive quantitative research based on content analysis
Source: PLoS One. 2024 Mar 7;19(3):e0298601. doi: 10.1371/journal.pone.0298601 (PMC10919601; doi:10.1371/journal.pone.0298601)
Supplement: S1 Data — (ZIP) [file pone.0298601.s001.zip › Data/地级市Z_F工作报告.zip!/║╙─╧/║╫▒┌╩╨/└┤╫╘╣1⁄2╓┌║┼í╛┬φ┐╦╩2╛▌═°í┐_2009.docx]

各位代表：

　　现在，我代表市人民政府，向大会报告过去五年的工作，对今后五年、重点是今年工作提出建议，请予审议，并请各位政协委员提出意见。

　　一、过去五年工作回顾

　　2004年以来，市政府在省委、省政府和市委的正确领导下，团结带领全市人民，认真贯彻落实科学发展观，深化改革、扩大开放，开拓进取、扎实工作，较好地完成了市八届人大确定的各项目标任务。

　　国民经济持续快速健康发展，综合实力显著提升。初步核算，2008年全市生产总值342亿元，比2003年增长1.1倍，年均增长16.4%；人均生产总值24070元，增加15903元，年均增长16.6%。全社会固定资产投资206亿元，五年累计完成602亿元，比上个五年增长4.4倍。规模以上工业增加值190亿元，年均增长24.2%。粮食单产连续5年创历史最高水平，总产连续3年突破100万吨。第三产业增加值75亿元，年均增长13.4%。三次产业比例调整为12.4︰65.8︰21.8。财政一般预算收入15.9亿元、支出32.4亿元，分别增长2.6倍和2.2倍。金融机构各项存款余额206亿元、贷款余额163亿元，年均分别增长16.7%和13.4%，域外金融机构贷款余额46亿元，初步形成了市内外金融机构合力支持经济社会发展的良好局面。

　　重点项目建设取得突破性进展，产业结构进一步优化升级。共安排重点项目294个，累计完成投资248亿元。竣工投产重点项目128个，其中超亿元项目78个，电厂二期三期、热电联供、盘石头水库、水泥厂二期三期等一批重点项目建成并发挥效益。在建重点项目28个，其中超亿元项目25个，煤化工项目一期顺利推进、二期开工建设，为谋求我市煤化工产业在全国同行业中的优势地位打下了基础。重点项目的强力推进，使我市大企业数量明显增多、规模明显扩大，规模以上工业企业由2003年的250家增加到409家，年销售收入超5亿元的企业由两家增加到13家；产业实力明显增强，煤电化材一体化、食品加工、机械制造三大战略支撑产业和金属镁深加工、光伏两大新兴先导产业等快速发展，被省确定为煤化工、汽车零部件、食品等产业基地。加快农业产业化经营和农畜产品加工业发展，年销售收入超亿元的龙头企业由两家增加到10家；畜牧业产值43亿元，增长1.5倍，占农业总产值的比重达到56.9%；食品工业增加值43亿元，增长3.2倍。一批现代服务业项目建成投用，被省确定为服务业改革综合试点市。旅游业取得新进展，成功创建国家4A级旅游区3个。宝山、金山、浚县和鹤淇产业集聚区被纳入省首批产业集聚区规划。

　　城市形象和品位明显提升，城乡面貌持续改观。市区建成区面积达到53平方公里，比2003年增加14平方公里。城镇化率达到47.8%，提高6.8个百分点。按照“城乡统筹、全域规划”原则，高标准高质量修编了城市总体规划，新区规划面积增加到65平方公里。新区建成区面积从2003年的13平方公里拓展到27平方公里。教育、文化、卫生等一大批社会事业项目建成投用，城市绿化覆盖率达到39.1%。老区一批基础设施、商业设施、公用事业等项目建成投用，城市功能进一步增强，人居环境得到改善。城中村改造取得新进展。两县县城加快道路、绿化等基础设施建设和住宅区开发，建设管理水平得到提高。加快基础设施、社会事业向农村延伸覆盖，农民生活生产条件进一步改善。建设农村公路2013公里，实现了所有行政村通油路、每个乡镇有一个二级以上公路出口，客运班车通达率99%。解决了31.4万人安全饮水问题。农村沼气用户达到宜建农户的50%。85%以上的行政村建有农村超市。在全国率先实现了广播电视村村通。完成了81个贫困村整村推进扶贫任务，2.85万农村贫困人口脱贫和解决温饱。

　　循环经济和节能减排工作扎实推进，生态环境质量保持良好。总投资80亿元的63个循环经济重点项目顺利实施，循环经济试点园区和试点企业建设取得明显成效，被确定为全国循环经济试点市、循环经济标准化试点市。建立完善节能降耗目标管理体系，积极推进重点节能项目建设；城区新建建筑节能覆盖率达到100%，被确定为全国建筑节能示范市。建成投用了4个污水处理厂、3个垃圾处理场等一批重点环保基础设施，市区污水处理率、垃圾无害化处理率分别达到85%、75%。取缔、关闭污染严重企业192家，河流出境断面水质平均达标率、环境空气质量优良天数达标率保持较高水平，城市饮用水源地水质达标率保持100%。淇河生态保护开发利用全面推进，新区段河道疏浚减漏治理、淇水诗苑、滨河乐园、淇河森林公园、淇河湿地公园、淇河植物公园“一河五园”工程建设正式启动。加快林业生态市建设，完成造林绿化54.6万亩，森林覆盖率提高5.6个百分点。

　　各项改革不断深化，对外开放实现重大突破。全面完成了国有企业改制工作，企业活力和市场竞争力明显增强。深化行政审批和收费制度改革，精简行政审批事项比例达到26%，取消、停止、降低标准行政事业性收费比例达到70%，取消、降低标准经营服务性收费比例达到85%。新区与老区、市区与县城统筹协调发展，开发区和淇滨区行政管理体制进一步理顺，浚县钜桥镇整建制划归淇滨区，为城市建设管理增添了活力和动力。乡镇机构改革顺利完成，农村综合改革扎实推进。市政公用事业经营管理体制等改革不断深化。市商业银行、邮政储蓄银行成功组建，农村信用社改革取得积极进展。坚持把招商引资作为经济工作重中之重，引进市外直接投资355亿元，年均增长69.2%；实际利用境外资金2.5亿美元，年均增长52.8%。成功引进美国维恩克、泰国万浦等一批外资企业。天海集团成功上市，实现了我市上市公司零的突破。非公有制经济增加值占生产总值的比重预计达到60%，比2003年提高15.8个百分点。

　　创新型鹤壁建设加快推进，核心竞争力进一步增强。科技综合实力进入全省先进行列。25项国家重大科技项目顺利实施；两项省重大科技专项落户我市，实现了承担省重大科技专项零的突破。15亩超高产攻关田夏玉米亩产创世界纪录，同一地块一年小麦、夏玉米两熟平均亩产创全国纪录；万亩夏玉米核心区单产创全国纪录。企业自主创新、引进消化吸收再创新能力进一步增强，国家和省级企业研发中心达到27家，省级以上创新型企业3家，首家博士后科研工作站挂牌运转。大力实施人才强市战略，引进急需高层次人才2000多名。实施职业教育发展攻坚计划，一批职业教育项目加速推进，现有职业学校发展势头良好。

　　民生得到持续改善，社会事业全面进步。累计投入财政资金25亿元，办成了一大批惠及人民群众的实事。城乡居民生活水平显著提高。城镇居民人均可支配收入12491元、农民人均纯收入5495元，年均分别增长15.9%和16.6%。社会消费品零售总额比2003年增加37亿元，年均增长17.3%。就业再就业工作成效明显。城镇新增就业20.8万人，下岗失业人员再就业9.3万人，近三年农村劳动力转移就业保持在20万人以上，被确定为全国统筹城乡就业试点城市、创业实训试点城市和创建创业型试点城市。社会保障水平持续提高。企业养老保险、城镇职工基本医疗保险参保人数分别达到13.8万人和20.6万人，比2003年增加2.3万人和5万人；城乡低保覆盖人数分别达到5.6万人、3.5万人；农村五保集中供养率由20%提高到40.8%；探索建立健全城乡困难群众救助体系，被确定为全国城乡救助体系建设示范市。采煤沉陷区综合治理、鹤煤公司棚户区改造累计完成投资22亿元，22480户市民居住条件得到根本改善。超前谋划推进的、涉及9319户的老城区棚户区改造工程正式启动。社会事业全面发展。全面实行城乡免费义务教育，对所有农村贫困学生免费提供教科书；建立了新的家庭经济困难学生资助体系，向高等中等职业教育学校学生发放奖助学金3967万元；改造农村中小学危房20万平方米，城乡办学条件明显改善；积极推进城乡教育均衡发展，教育质量得到新提升。市文化中心、农村示范性文化大院等一批文化事业、文化产业项目建成投用，一批优秀精品剧目荣获国家级奖项，成功举办首届中国鹤壁民俗文化节，广泛开展广场文化艺术节等多种形式的文化活动，丰富了群众精神文化生活。深入开展全民健身活动，建成22处社区、乡镇体育活动场所和66个农民体育健身工程。新型农村合作医疗基本覆盖农村居民，城镇居民医疗保险试点工作顺利推进，在全省率先基本实现每个行政村有一所标准化卫生室，城乡居民看病难、吃药贵问题得到缓解。建成32个公共卫生项目，提高了重大传染病防控和应对突发公共卫生事件能力。在全省率先推行农村部分计划生育家庭社会养老保险，人口自然增长率保持在5%。以下，稳定了低生育水平。

　　民主法制建设进一步加强，精神文明建设取得新进展。坚持依法行政和依法治市，自觉接受人大及其常委会的监督，支持政协参政议政，认真办理人大代表建议和政协提案1270件，办结率100%。政务公开工作深入推进。第六届村委会换届顺利完成。扎实推进以转变政府职能、转变工作作风、提高行政效能、提高公务员素质为重点的“两转两提”工作，全面建成市县乡三级行政服务网络，开通“12345”市长热线、市长信箱、政府网站留言板等服务平台，加强了政府与企业、与群众的沟通交流，及时解决了企业和群众反映的问题。勤政廉政和政风行风建设、财政和审计监督等工作取得新成效。扎实推进信访工作，多次被评为全省信访工作先进市。刑事、治安案件连年大幅下降，连续5年实现现行命案全破，侦破率保持全省第一，连续4年被评为全省平安建设先进市。切实加强安全生产工作，安全事故起数、死亡人数连年大幅下降。加强监督管理和专项整治，食品药品质量安全水平进一步提高。国防和军队建设力度继续加大，一些军事设施开工建设。双拥共建活动扎实推进，军政军民团结不断加强。社会主义荣辱观教育和精神文明创建活动深入开展，涌现出程相文、李飞等一大批先进典型人物，全社会文明程度明显提高，广大干部群众的精神风貌发生可喜变化。外事、侨务、对台、民族、宗教、统计、档案、史志、气象、地震等工作取得新成绩。四川抗震救灾的救援和过渡房建设任务圆满完成。

　　各位代表！

　　过去的五年是我市发展历史上不平凡的五年。全市经济持续发展之快前所未有，城乡面貌变化之大前所未有，人民群众得到实惠之多前所未有，鹤壁综合实力和整体形象显著提升，进入了新的发展时期。尤其是2008年，受国际金融危机影响，第三季度以来经济下行压力逐渐加剧，在这样的情况下，煤化工等一批重点项目顺利推进，全市生产总值、全社会固定资产投资、规模以上工业增加值、财政收入等主要经济指标，仍然实现两位数增长。过去五年特别是2008年的成绩来之不易，这是省委、省政府和市委正确领导的结果，是市人大、市政协大力支持的结果，是全市人民共同奋斗的结果。在此，我代表市人民政府，向全市人民，向各民主党派、各人民团体和各界人士，表示诚挚的感谢！向关心、支持我市现代化建设的同志们、朋友们，表示诚挚的感谢！

　　五年来，我们始终坚持用科学发展观统领经济社会发展全局，紧紧抓住发展第一要务不放松，牢牢扭住项目建设主抓手不动摇，正确处理工业与农业、新区与老区、经济与民生、发展与生态等关系，做到既突出重点又协调推进，力求使发展更迅速、更全面、更科学。坚持靠解放思想、改革开放增强发展动力和活力，不断更新观念、明晰思路、创新体制机制，注重用改革发展的理念解决前进中的困难和问题，深入实施开放带动战略，超常规推进各项工作，调动方方面面的积极性、创造性。坚持以超前谋划、真抓实干争取工作主动，敏锐把握国内外大势，认真谋划事关长远发展的战略思路和构想，高质量高效率推进每一项工作、抓好每一个环节，为我市在激烈的竞争中加快发展赢得宝贵先机。坚持把以人为本、改善民生作为发展的出发点和落脚点，集中力量，加大投入，扎扎实实解决人民群众最关心最直接最现实的利益问题，让改革发展的成果惠及广大人民群众。

　　我们也清醒地看到，当前我市经济社会发展还存在一些不容忽视的矛盾和问题，主要是产业规模小、层次低等深层次问题尚未得到根本解决，资源开发利用的效益和水平有待进一步提高，第三产业发展与经济整体发展还不协调，农民持续增收难度较大；外部形势对我市经济发展的负面影响仍较突出，部分行业和企业生产经营困难，经济下行和财政保障压力加大；社会事业发展存在薄弱环节，一些关系群众切身利益、影响社会和谐稳定的问题亟待解决；政府职能转变还不到位，个别政府工作人员的责任意识、大局观念、工作作风、服务水平与新形势新任务和广大人民群众的要求还存在差距。

　　我们要深刻认识面临形势和问题的严峻性、复杂性，增强危机意识和忧患意识，充分利用有利条件，努力做好各项工作，不辜负人民群众的期望和重托。

　　二、今后五年奋斗目标和2009年总体要求

　　今后五年，是我市抢抓战略机遇，迅速培育壮大产业支撑、加快经济结构调整和发展方式转变、不断增强整体竞争实力，加速工业化、城镇化、农业现代化进程的关键时期。我们要以科学发展观统领经济社会发展全局，进一步唱响率先崛起主旋律，加快构建现代产业基础和现代城市骨架，加快建设文化强市、和谐鹤壁、生态鹤壁，努力保持经济社会又好又快发展，尽快使我市工业强起来、农业富起来、三产发起来、生态美起来！

　　——现代产业框架基本形成。全市三大战略支撑产业由比较优势转变为竞争优势，两大新兴先导产业培育壮大为新的经济支柱，部分产业在全省乃至全国占有重要位置。4个产业聚集区建成各具特色的规模产业集群，力争宝山循环经济园区成为全国示范性循环经济园区，我市成为全国循环经济示范市。巩固粮食总产、提高单产，加快畜牧业发展，保持在全省的先进位次。加速提升服务业发展水平和占国民经济的比重，三次产业发展更趋协调。力争全社会固定资产投资年均增长25%以上，生产总值保持两位数增长，财政状况明显好转，综合经济实力显著提升。

　　——现代城市骨架基本形成。以城市综合服务功能区建设为龙头，带动新区建设全面升级，65平方公里主框架基本拉开。老区和县城改造建设加快推进，功能更加完善，品位明显提升。鹤淇一体化及“一核双星”单元组合型现代城市格局基本形成，城镇化率接近60%，继续保持全省先进位次，城市载体功能、辐射能力、带动作用明显增强，开放型、创新型、宜居型特色更加凸现，城乡经济社会发展一体化格局基本形成。

　　——文化强市建设更具特色。鹤壁核心文化价值观的引领作用得到充分发挥，文化资源优势得到有效挖掘，文化事业全面繁荣，特色文化产业快速发展，军事文化、民俗文化、诗文化等成为全国知名文化品牌，“两山一水一节”成为全国知名旅游品牌。基层公共文化设施全面改善，公共文化产品服务和供给能力显著提升。科技创新、职业教育发展等走在全省前列，创新型鹤壁建设取得重大突破。城乡居民思想道德素质、科学文化素质和健康素质不断提高，城市综合竞争软实力明显增强。

　　——和谐鹤壁建设更臻全面。贯彻落实科学发展观的体制机制不断健全。城镇居民人均可支配收入跨入全省先进行列，农民人均纯收入保持全省先进位次。基本公共服务趋于均等化，城乡义务教育均衡发展，基本医疗卫生服务不断改善，劳动就业更加充分，覆盖城乡居民的社会保障体系全面建立，民主法制建设扎实推进，平安建设水平显著提升，人民群众的幸福感不断增强。

　　——生态文明建设更富成效。淇河自然生态风貌得到恢复，古韵文明得到进一步挖掘弘扬。能源资源利用效率大幅提高，主要污染物排放量得到有效控制，生态环境质量全面改善，城乡面貌明显改观，经济发展与人口、资源、环境趋于协调，生态城市基本建成。资源节约型、环境友好型的产业结构、增长方式和消费模式初步形成。

　　2009年政府工作的总体要求是：深入贯彻落实科学发展观，紧紧围绕加快“两个构建”、实现“两保”目标，坚持解放思想不停步，坚定发展信心不动摇，积极在应对金融危机中抢抓发展机遇，以强化项目建设、扩大投资需求为重点，优化产业结构、转变经济发展方式；以深化改革、扩大开放为动力，增强经济社会发展活力；以加强社会建设、着力改善民生为出发点和落脚点，认真解决涉及群众利益的难点热点问题；以提高服务水平、优化发展环境为保障，努力保持经济社会又好又快发展。

　　经济社会发展的主要目标是：生产总值增长11%左右；全社会固定资产投资增长23%；规模以上工业增加值增长15%；财政一般预算收入增长7%左右；社会消费品零售总额增长16%；引进市外直接投资确保90亿元，力争100亿元；城镇居民人均可支配收入实际增长7%，农民人均纯收入实际增长6%；单位生产总值能耗降低5.1%，化学需氧量、二氧化硫排放量分别下降3.8%和5%；人口自然增长率控制在4.7%。以内；城镇新增就业3万人以上。

　　三、2009年主要任务

　　当前，我市经济社会发展基本面是好的，应该充分肯定。同时也要看到，国际金融危机尚未见底，对实体经济的影响还在蔓延，全球经济增长减速、我国经济周期性调整、我市经济下行与产业竞争力不强的矛盾和问题叠加重合，我们面临的形势是严峻的，应该高度重视。但是从总体上看，我国经济社会发展的基本面和长期向好的趋势没有改变，特别是国家实施积极的财政政策和适度宽松的货币政策，为我们加快基础设施建设等提供了难得机遇；部分地区、行业发展和企业生产经营已出现回暖迹象，一些优势企业逆势而上、加快调整产品产业布局，金融危机形成的倒逼机制、区域竞争格局重塑，为我们加快引进项目、优化产品产业结构提供了有利条件；全市上下团结协作、拼搏进取、干事创业的精神风貌，为我们直面挑战、化危为机、加快发展提供了坚强保证，应该坚定信心。

　　围绕政府工作总体要求和奋斗目标，今年要着力抓好以下几个方面的工作：

　　(一)坚持把项目建设作为各项工作主抓手，努力在现代产业基础构建上取得新突破

　　加快建设一批事关全市长远发展的大项目，进一步做强战略支撑产业、做大新兴先导产业、创建节能环保产业，加快构建现代工业、现代农业、现代服务业体系。

　　坚持不懈抓好项目建设。把今年作为“项目建设年”，坚持围绕项目建设、服务项目建设、有利项目建设，继续采取超常规推进措施，力争使今年成为我市落地项目最多最好、项目建设速度最快、完成投资额最大的一年。着力抓好70个重点项目，力争完成投资115亿元以上，其中煤化工一期、二期等38个工业项目完成投资68.5亿元，鹤辉高速、鹤淇大道等19个基础设施、社会事业项目完成投资21亿元，粮食物流园区等3个商贸流通项目完成投资2.5亿元，盘石头水库移民工程等5个农业项目完成投资2亿元；力争21个项目竣工投产。做好6个过境项目的协调服务，确保在我市顺利建设。重点推进煤化工后续、盐化工等总投资378亿元的43个项目前期工作，力争22个开工或达到开工条件。

　　进一步加大招商引资和争取上级资金力度。突出重点地区、重点产业、重点企业，着力引进产业关联度高、核心竞争力强的行业龙头企业及产业集群，力争使引进的企业和项目以组团形式整体落地。市、县区两级财政安排专项资金4000万元，用于项目的谋划推进，力争新策划超亿元项目40个；引进超5000万元工业项目40个，其中超亿元项目20个。瞄准中石化、河南煤化集团等大企业大品牌，积极寻找战略合作伙伴，推动企业战略重组与合作。认真分析、准确把握我市发展与国家、省战略层面的结合点，严格目标责任制和奖惩机制，力争在全省争取国家扩内需、保增长资金中所占比例达到1/18。

　　努力为企业排忧解难。扎实开展“企业服务年”活动，因企制宜、分类指导，突出重点、带动一般，努力解难题、办实事、促发展。认真落实各项涉企优惠政策，切实减轻企业负担。积极引导企业加强协作，不断提高抗风险能力。对发展快、贡献大的企业实施奖励。加强组织协调和科学指导，着力解决企业遇到的实际问题，满腔热情地帮助企业加强基础管理、提高战略管理水平，保障生产要素供给，推动产品升级换代，积极扩大发展规模，与企业同舟共济、共克时艰。

　　大力发展现代农业。认真落实强农惠农政策，切实抓好粮食生产，充分发挥自身优势，促进农业稳定发展和农民持续增收。加快建设优质小麦、玉米生产基地，着力建好5个万亩高产攻关示范区，争取实施万亩连片小麦玉米高产核心示范区，发展壮大50个规模经营粮食生产合作组织，争取培养500家种植面积超百亩、产粮超百吨的种粮大户。大力发展林业、瓜果、花卉等生态休闲和高效农业，抓紧推进生态观光农业园区建设。加快发展现代畜牧业，建设、改造20个年出栏1万头和10个年出栏5万头的标准化生态环保养猪场，30个年出栏60万只的标准化肉鸡场，5个年存栏500头的标准化奶牛养殖场。积极培育优质农畜产品生产加工基地，加快推进、争取签约落地一批超5000万元的食品工业项目。积极打造农业产业化龙头企业集群，大力发展农民专业合作组织，进一步提高农业产业化水平。着力抓好农业综合开发、优质粮食产业工程等重大基础设施建设项目，加强耕地和基本农田综合整治，提高农业综合生产能力。加强新型农业科技推广体系建设，完善农产品质检体系和动物疫病防控体系。

　　加快发展服务业。高起点、高标准编制服务业发展总体规划和专项规划，制定服务业发展意见和政策措施，扎实开展服务业综合改革试点工作。着力推进一批支撑能力强、带动产业升级效果明显的服务业项目，加快培育壮大一批服务业重点企业。抓好住宅小区等房地产项目，引导房地产业稳定健康发展。注重运用先进理念、现代技术发展提升商贸、餐饮等传统服务业，努力引进大型商业连锁企业。积极发展文化产业、商贸物流、科技服务等特色服务业，为现代工业和现代城市发展提供有力支撑。

　　下大力气优化发展环境。坚持重点产业特别政策、重点企业特殊服务，最大限度地降低建设和生产经营成本，严肃查处破坏发展环境的案件事件，使项目和企业招得来、稳得住、发展好。以市行政服务中心为依托，建立项目审批绿色通道，更好地实行外来投资项目代办制。实行招商引资项目副县级以上干部联系分包制度，帮助企业解决项目建设、生产经营遇到的困难和问题。设立产业发展基金1000万元，对重点项目予以贷款贴息，对按节点计划投资或提前完成投资的项目责任单位给予奖励。建立重点项目定期报告、每月通报、每季度巡回观摩等制度。组建精干高效的产业集聚区管理服务机构，加大工作推进力度，加快集聚区基础设施建设和招商引资工作，尽快开工建设一批关联度高、发展性好、竞争力强的项目。

　　(二)加快推进城乡一体化，努力在现代城市骨架构建上取得新突破

　　按照建设单元组合型城市的思路，坚持城乡统筹、协调发展，强化新区、优化老区、区县联动，全面提升城市形象和品位，城镇化率力争提高两个百分点。

　　加强城乡规划工作。坚持前瞻性、科学性的原则，高标准完成城乡总体规划修编，同步完成土地利用总体规划、产业集聚区规划和矿产资源规划修编。搞好城市综合服务功能区、职业教育园区的控制性详细规划。指导县区修编城市总体规划、建设发展规划、村镇体系规划等。抓好规划的宣传、引导和执行，维护规划的权威性、严肃性。

　　加强城市基础设施建设。以城市综合服务功能区建设为重点，掀起城市建设新高潮。抓好滨河路、朝歌路、渤海路等城市道路建设，进一步拉大城市框架。加快推进快速通道向北延伸工程。启动棉丰渠改造等项目，搞好城市“三纵四横”水系建设，进一步提升城市品位。抓好市体育中心、艺术中心、金融大厦等精品工程建设，进一步增强城市功能，提升城市形象。建成投用新区“两小一初”3所学校。高度重视老区和两县县城发展，加快老区改造和淇县城北新区、浚县国家历史文化名城建设。出台完善有关政策，下大力气推进城中村改造。深化供热体制改革，扩大供水、供气能力，完善城市公交设施，加快发展公用事业。积极推进农村居民向城镇聚集，做好乡改镇等工作。拓宽城市建设投融资渠道，逐步建立政府引导、市场运作的多元化、多渠道城市建设投融资体制。

　　加强城市管理。大力开展城市环境综合治理和生态环境改善工作，提高人性化、规范化、科学化、精细化管理水平。启动道路整修、绿化完善、部分建筑装饰等建成区改造提升项目，抓好城市背街小巷和城乡接合部的环境卫生。加强社区管理工作，增强物业服务能力，提升社区管理服务水平。

　　加强新农村建设。增加并整合支农资金，加快农村基础设施建设。新建、改建农村公路150公里、桥梁10座。解决7万农村居民饮水安全及山区群众饮水困难问题。完成15个贫困村整体推进扶贫开发。实施“百村万户”通沼气工程，新增户用沼气1.3万户，农村沼气用户达到宜建农户的63%以上。探索建立改善村容村貌长效机制，完成150个村镇绿化工程，创建10个省级生态文明村。进一步搞好新农村建设试点工作，促进市县乡三级试点村协调发展。深化农村综合改革，增强乡镇政府社会管理和公共服务能力。加强土地承包经营权流转管理和服务。完成集体林权制度改革。探索新型农村社区建设途径和办法，启动农民集中居住区建设试点工作。

　　(三)积极推进文化发展和精神文明建设，努力在文化强市建设上取得新突破

　　联系实际，突出特色，推动文化发展与繁荣，提升文化软实力，为实现率先崛起提供思想保证、舆论支持和文化支撑。

　　大力发展文化事业。在充分发挥现有文化设施作用的同时，完成22个乡镇综合文化站扩建、50个农家书屋和30个农村示范性文化大院建设，扶持建设673个农村扩展型文化信息资源共享工程基层服务网点。举办庆祝新中国成立60周年系列文化活动和第二届广场文化艺术节，继续办好中国鹤壁民俗文化节。加强文物和非物质文化遗产保护。建成58个农民体育健身工程，开展以“全民健身日”为主题的系列体育活动。大力发展农村有线电视，加快广播电视数字化步伐。

　　加快发展文化产业。尽快制定实施文化旅游产业发展规划。加大以淇河为龙头的旅游开发和宣传推介力度，努力培育文化旅游、文化创意、文博会展等产业，提升旅游区档次和水平，提升文化旅游业竞争力。积极推进淇水诗苑、民间工艺品会展等一批文化产业项目。加快建设浚县石刻泥塑特色文化产业区块，大力发展特色文化产业集群。打造文化精品工程，发扬光大牛派艺术。繁荣、规范文化市场，加强网络文化建设和管理。积极稳妥推进文化体制改革。

　　深入开展精神文明建设。加强社会主义核心价值体系建设，在全社会树立中国特色社会主义共同理想，大力弘扬以爱国主义为核心的民族精神和以改革创新为核心的时代精神。启动鹤壁核心文化价值体系建设工程，探索挖掘核心文化标识，塑造鹤壁独特的城市灵魂和共同的精神追求。认真开展深入学习实践科学发展观活动，提高干部群众推动科学发展的能力和水平。深入开展“知荣辱、讲正气、促和谐”主题道德实践活动。加强公民意识教育。做好大学生和未成年人思想政治工作。强化诚信体系建设。大力开展以优美环境、优良秩序、优质服务和创建文明城市、文明社区、文明景区为主要内容的“三优三创”竞赛活动，以“清洁家园行动”为载体的农村精神文明创建活动，继续推进文化科技卫生“三下乡”，推动群众性精神文明创建活动蓬勃开展，提高城乡文明程度。

　　(四)扎实推进资源节约型和环境友好型社会建设，努力在生态城市工程建设上取得新突破

　　把生态建设放在与产业发展、城市建设同等重要的位置抓紧抓好，让人民群众更好地享受生态文明。

　　进一步提升循环经济发展水平。强力推进循环经济项目建设，力争18个续建项目全部投产，12个新开工项目加快推进，27个谋划项目取得实质性进展。规划建设产业集聚区废弃物综合利用项目。推进循环经济和环保制造业园区建设。加快循环经济技术支撑体系建设。初步建立循环经济标准化体系，努力为全国循环经济发展提供依据。全面铺开城市再生资源回收利用体系建设，开展低碳社区试点工作。

　　强力推进节能降耗工作。突出抓好工业、交通、建筑等领域节能工作。深入推进重点耗能企业节能行动，将年度综合能耗5000吨标煤以上的企业全部纳入市级动态监管，争取一批企业能效指标达到国内或国际先进水平。加快重点节能项目建设，争取全年节能32万吨标煤。启动工业锅炉、电机系统节能改造专项计划，推广绿色照明工程。推动城市集中供暖分户控制和用热计量收费工作，争创全国可再生能源应用示范城市。完善监管机制，提高节能降耗监管能力。积极推动土地、矿产资源节约集约利用。启动乡镇禁用实心黏土砖、农村节能房屋建设试点工作。

　　毫不松懈地加强污染减排和生态建设。严格实行主要污染物总量控制。深化工业污染防治，实施重点涉水工业点源深度治理。加强卫河流域环境综合整治和农村面源污染防治。严把环保准入关，保持新建、改建、扩建项目环评执行率和“三同时”率100%。强化环境自动监控系统建设，完成城市污水处理厂化学需氧量和氨氮自动监控设备更新改造，确保建成的污水处理厂全面稳定达标运营，燃煤电厂脱硫设施投运率达到95%以上。适当提高排污费、污水和垃圾处理费征收标准，完成山城区垃圾填埋场封场治污工作。鼓励企业加大环保投入，完善环保设施。以浮山森林公园、淇河森林公园、淇河新区段两岸生态恢复、宝山林业生态园区等为重点，大规模高质量开展造林绿化，加快林业生态市建设。

　　(五)以科技创新、人才培养引进为重点，努力在创新型鹤壁建设上取得新突破

　　着眼当前和长远发展，加快构建科技创新和人才支撑体系，积极创造跨越发展、率先崛起新优势。

　　进一步提高科技创新能力。坚持重点在引进、关键在应用、企业为主体的原则，加强产学研结合，努力破解制约重点产业发展的关键技术，切实增强自主创新能力和核心竞争力。认真组织实施科技创新三年计划和“十一五”国家粮食丰产科技工程、国家科技支撑计划项目、省重大科技专项。整合科技资源，积极打造科技创新平台，加快筹建鹤壁农科院，强力推进科技服务中心建设，新增省级企业研发中心6家以上。实施企业创新能力培育科技工程、科研成果转化工程。提高知识产权创造、运用、保护和管理水平。

　　大力发展职业教育。加快实施职业教育攻坚计划，扎实推进职业教育园区、职业技术学院新校区、与中国电子电路印制协会联合办学、鹤煤技师学院扩建等项目建设，积极引进河南科技学院新科学院等来鹤办学，参与河南豫北高等职业教育集团组建，努力实现职业教育跨越式发展。对初中、高中毕业后未升学人员进行全员职业技能培训。加快发展农村职业教育并逐步实行免费。继续实施三年人才引进计划，积极引进急需高层次人才，切实用好现有人才，更好地留住人才。

　　(六)着力解决涉及群众利益的难点热点问题，努力在民生改善上取得新突破

　　越是困难情况下，越要注重保障和改善民生，尤其是解决好基层困难群体的生活问题。以落实好省委省政府“十大实事”、办好我市12项民生工程为重点，认真为群众办实事、办好事。

　　努力扩大社会就业。实施更加积极的就业政策，多渠道增加就业岗位。发挥政府投资和重大项目带动就业的作用，提高服务业、劳动密集型产业、中小企业、非公有制经济吸纳就业的能力。大力支持自主创业、自谋职业，促进以创业带就业。进一步开发公益性岗位，继续延长“4050”人员享受公益性岗位就业的各项补贴政策，动态解决“零就业家庭”就业问题。鼓励和支持困难企业与员工协商薪酬，采取灵活用工、弹性工时、技能培训等办法，尽量不裁员。落实农民工返乡创业的政策措施，促进农民工就业。下岗失业人员再就业8100人以上。农村劳动力转移就业24万人以上。做好高校毕业生和复转军人就业安置工作。

　　进一步提高社会保障和居民收入水平。做好养老保险扩面征缴工作，做实企业职工基本养老保险个人账户，确保全市企业退休职工养老金按时足额发放。积极探索农村居民养老保险工作，全面实施被征地农民社会保障制度。完善失业保险制度，确保失业保险金按时足额支付。实现新农合网络化动态监管，农民参合率稳定在95%以上；做好城镇居民基本医疗保险工作，城镇居民参保率稳定在80%以上。积极推行城乡居民医疗救助制度。完善城乡社会救助体系和优抚保障制度，大力发展社会福利和慈善事业。增加对高等院校家庭经济困难学生的生活补助。提高企业退休人员基本养老金水平，提高优抚对象、建国前老党员等抚恤和生活补助标准。适当提高城乡低保保障标准，巩固提高农村五保集中供养率，农村五保集中供养标准由每年不低于1400元提高到不低于1600元。实施蓄滞洪区安全建设工程。建成儿童救助保护中心。开工建设残疾人综合服务中心。努力创建全国无障碍建设城市。加快推进老城区棚户区改造工程。建设廉租住房5.28万平方米，提高廉租住房补贴标准，发放廉租补贴4520户。推进事业单位工资制度改革，落实企业最低工资制度，健全农民工工资支付保障机制。

　　大力发展教育事业。落实城乡免费义务教育政策，巩固提高义务教育，提高农村中小学生人均公用经费标准。加强教师队伍建设，提高中小学教师待遇。认真解决农民工子女在输入地免费接受义务教育问题。推进素质教育，让学生有更多时间思考、实践和创造。巩固农村中小学实验室改造成果，提高中小学多媒体设备应用水平。搞好城乡中小学校舍维修改造，确保学生安全。

　　加快完善医疗卫生服务体系。坚持公共医疗卫生的公益性质，加快建立覆盖城乡的基本医疗卫生制度。加快推进基本医疗保障制度建设，健全基层医疗卫生服务体系，促进基本公共卫生服务均等化。完成7所乡镇卫生院改扩建任务。对农村标准化卫生室实施规范化管理，实现社区卫生服务中心(站)全覆盖，提升服务能力和水平。积极创建“白内障无障碍市”，为全市白内障手术适应症患者全部免费实施复明手术。加强人口和计划生育工作，综合治理出生人口性别比偏高问题，完善计划生育利益导向机制，农村部分计划生育家庭奖励扶助标准由每人每年600元提高到840元，稳定低生育水平。

　　全面提高产品质量和安全生产水平。把人的生命安全放在至高无上的位置，认真开展整顿规范市场秩序专项行动及“质量和安全年”活动，深入开展食品药品安全专项整治，实行严格的食品安全质量追溯、召回、市场准入和退出制度，让群众买得放心、吃得安心、用得舒心。扎实开展“安全生产年”活动，全面落实安全生产责任制。做好小煤矿整顿整合和关闭取缔工作。深入开展安全生产隐患排查治理，着力抓好重点行业、重点企业安全管理和公共聚集地安全防范，坚决遏制重特大安全事故发生。

　　全力维护社会和谐稳定。高度重视维护群众合法权益，及时合理解决群众反映的问题，引导群众以理性合法的方式表达利益诉求。强化源头治理，有效防范和妥善处置征地拆迁、涉法涉诉、农民工工资、企业破产、环境污染等可能引发的群体性事件，确保社会治安形势、信访形势和安全形势基本平稳。开展法治城市创建活动，做好法律援助应援尽援工作。深入开展平安创建活动，加强社会治安综合治理，严密防范、依法严厉打击各类违法犯罪活动，增强人民群众安全感。支持工会、共青团、妇联等人民团体充分发挥密切联系群众的桥梁纽带作用，参与社会事务管理。深入开展国防教育，支持驻鹤解放军和武警部队建设，开工建设民兵综合训练基地，不断提高部队、民兵预备役和人民防空建设质量；积极开展双拥共建活动，巩固和发展军政军民团结。认真搞好第二次经济普查等国情国力调查。做好外事、侨务、对台、民族、宗教、档案、史志、气象、地震等工作。按期完成支援四川江油市东安乡、河口镇灾后重建任务。

　　(七)切实做好财税金融工作，努力在支持经济社会跨越发展上取得新突破

　　认真落实财政、金融政策，坚持发挥市场作用和政府调节两手抓，努力办大事、破难题、促发展。

　　全面做好财税工作。依法加强税收征管，规范非税收入管理，推进社会化、多元化融资，为全市发展提供有力的财力支持。牢固树立过紧日子的观念，大力发扬勤俭节约精神。优化支出结构，对公务购车用车、会议、公务接待、办公设备、出国出境等经费支出实行零增长，确保法定支出、工资发放、改善民生、维护稳定、落实重点项目配套资金等需要。综合运用多种手段，扩大政府投资，引导带动社会投资。坚持节约集约原则，科学调度，加强监管，充分发挥财政资金的经济社会效益。

　　更好发挥金融的积极作用。创新和改进金融服务，满足合理资金需求，促进经济社会发展。通过定期召开银企洽谈会等形式，及时解决金融机构与企业合作中存在的问题。加强金融生态环境建设，鼓励银行业金融机构在风险可控的前提下加大信贷投放力度，进一步加强与市外股份制银行合作。支持市城市商业银行在两县设立支行。加快组建农村合作银行。积极开展小额贷款公司试点工作。加快融资平台建设，创新融资方式，支持企业择机上市。加快中小企业信用担保体系建设，引导社会资本投资设立担保机构，力争全市担保资本金达到2.5亿元左右。

　　四、加强政府自身建设

　　继续以“两转两提”为重点，深入推进服务型政府建设，努力提高驾驭经济社会发展的能力。

　　全面推进依法行政。严格依照法定权限和程序履行职责、接受监督。严格执行人大及其常委会的决议、决定，自觉接受人大及其常委会的法律监督和工作监督；加强与人民政协的协商，自觉接受人民政协的民主监督；广泛听取各民主党派、工商联和无党派人士的意见和建议；强化监察、审计等专门监督；高度重视人民群众监督和新闻舆论监督。认真办理人大代表建议和政协提案。加强政府法制工作。深入推行政务公开。

　　加快转变政府职能。坚持发挥市场在资源配置中的基础性作用，与行政机关挂靠的中介机构全部推向市场。积极稳妥推进政府机构改革，争取上半年完成市政府机构改革，年底前完成县级政府机构改革。深化行政审批制度改革，进一步减少和规范行政审批事项，减少行政事业性收费和经营服务性收费，加强对已取消审批和收费事项的后续监管。健全政府绩效考核体系，把各方面工作引导到科学发展轨道上来。创新社会管理体制，加强基层基础工作。完善应急预案体系和管理体制，有效应对突发事件，提高危机管理和抗风险能力。加快市县电子政务建设应用，进一步提高服务水平。

　　切实加强廉政建设。以完善惩治和预防腐败体系为重点，加强反腐倡廉建设，严格落实廉政建设责任制，确保权力阳光操作、规范运行。加强对中央和省扩内需、保增长政策措施执行情况的监督检查，保证政策落实到位、取得预期效果。认真落实审批、规划等“十公开、十不准”规定，加强工程建设监管。严格执行领导干部廉洁自律规定，清清白白做人，干干净净做事。坚决查处腐败案件，依法惩处腐败分子。

　　进一步转变工作作风。认真贯彻执行上级决策部署，创造性地开展工作，做到摸实情、办实事、出实招、见实效。密切联系群众，关心群众疾苦，倾听群众呼声，为群众排忧解难。坚持科学民主决策，提高决策的预见性、科学性和有效性。进一步创新体制机制，精简优化工作程序，提高办事效率和服务水平。积极开展争做“人民满意的公务员”活动，努力建设政治坚定、业务精通、清正廉洁、作风优良的公务员队伍。始终保持昂扬向上、奋发有为的精神状态，和广大人民群众一道迎难而进，开拓进取，艰苦奋斗，共克时艰。

　　各位代表！

　　面对全市人民的期待，我们肩负的责任重大、使命光荣。让我们坚定必胜信心，勇敢面对挑战，不断解放思想，积极开拓创新，以更加开阔的视野、更加有力的措施、更加扎实的作风，努力在克服困难中开创跨越发展新局面，在战胜挑战中谱写率先崛起新篇章，在锐意进取中创造无愧于人民的新业绩，以优异成绩迎接新中国成立60周年！
